# Polyhydroxyalkanoate Production Under Saline Conditions: A Review of Pure and Mixed Microbial Culture Systems

**DOI:** 10.3390/polym18212682

**Published:** 2026-10-29

**Authors:** Alessandra Filieri, Sidra Yousaf, Chiara Morandi, Silvia Crognale

**Affiliations:** Department for Innovation in Biological, Agro-Food and Forest Systems (DIBAF), University of Tuscia, Via S. Camillo De Lellis, 01100 Viterbo, Italy; alessandra.filieri@unitus.it (A.F.); sidra.yousaf@studenti.unitus.it (S.Y.); chiara.morandi@unitus.it (C.M.)

**Keywords:** polyhydroxyalkanoates (PHAs), pure culture, mixed microbial culture, halophilic microorganisms, metabolic engineering

## Abstract

Polyhydroxyalkanoates (PHAs) are promising biodegradable and bio-based alternatives to conventional plastics; however, their industrial implementation remains challenged by high production costs, refined carbon sources, stringent cultivation requirements, and energy-intensive downstream processing. Saline bioprocessing offers an emerging strategy to address these limitations by exploiting the robustness of halophilic and halotolerant microorganisms and valorizing saline, low-cost, and waste-derived feedstocks. This review provides a critical analysis of PHA production under saline conditions, focusing on halophilic and halotolerant pure cultures and saline mixed microbial cultures (MMCs). The bioprocess chain is examined, encompassing feedstock selection and pretreatment, microbial adaptation, cultivation and enrichment, process optimization, metabolic and genome engineering, and PHA recovery and purification. Particular attention is given to physiological responses to salinity and their effects on growth, carbon allocation, PHA accumulation, and polymer composition. Pure cultures and MMCs are compared in terms of substrate flexibility, process robustness, productivity, polymer properties, scalability, and downstream requirements. Emerging strategies, including waste valorization, saline resource integration, community engineering, process control, and salt recovery, are discussed. Finally, knowledge gaps and technological bottlenecks are identified to guide the development of viable and sustainable saline PHA bioprocesses.

## 1. Introduction

Petroleum-based polymers are extensively used in industrial and consumer applications owing to their favorable physicochemical properties and relatively low production costs. However, their persistence and inadequate end-of-life management have resulted in the widespread accumulation of plastic waste in terrestrial and aquatic environments. If current production and waste-management trends continue, approximately 12 billion metric tons of plastic waste could accumulate in landfills or the natural environment by 2050 [1]. These concerns have stimulated increasing interest in bio-based and biodegradable polymers as potential alternatives to selected conventional plastics. PHAs are microbial polyesters that can serve as intracellular carbon and energy reserves. Their physicochemical properties can be tailored through their monomer composition, supporting potential applications in packaging, agriculture, and biomedical materials. Nevertheless, high production and downstream-processing costs continue to limit their widespread industrial implementation. PHAs are intracellularly produced by various bacteria and archaea through regulated metabolic pathways, resulting in polymers with diverse chemical compositions and material properties. Due to their biodegradability, biocompatibility, and tunable characteristics, PHAs are among the most extensively studied and commercially promising microbial polymers [2]. The current commercial producers of PHA include, among others, Danimer Scientific (Bainbridge, GA, USA), Shenzhen Ecomann Biotechnology Co., Ltd. (Shenzhen, China), Kaneka Corporation (Osaka, Japan), RWDC Industries (Singapore), TianAn Biologic Materials Co., Ltd. (Ningbo, China), Newlight Technologies LLC (Huntington Beach, CA, USA), and Biomer (Schwalbach, Germany). However, large-scale commercialization of PHA production remains constrained by relatively high production costs. Primary constraints include expensive sterilization processes, prevention of contamination. Additionally, the use of primary feedstock such as glucose presents economic and environmental concerns, including substantial water usage and costly downstream processing requirements [3]. Among the various approaches proposed to improve process sustainability and economic competitiveness, saline bioprocesses have attracted increasing attention. The use of halophilic and halotolerant microorganisms, as well as saline mixed microbial cultures, enables operation under high-salinity conditions, offering advantages such as reduced sterilization requirements, simplified downstream processing, and the possibility of exploiting saline waste streams and seawater as process resources. To improve process economics, research has increasingly focused on three complementary upstream strategies: (i) the discovery of robust halophilic and halotolerant PHA-producing strains from diverse environments, exploiting their natural adaptation to saline conditions and high PHA accumulation capacity [4]; (ii) the development of saline mixed microbial culture (MMC) systems capable of valorizing saline waste streams and other inexpensive feedstocks; and (iii) the application of metabolic engineering to improve microbial performance, substrate utilization, and PHA productivity [5,6,7,8].

This review provides an overview of microbial PHAs production under saline conditions, with particular emphasis on comparing halophilic and halotolerant pure cultures with saline MMCs. It critically discusses the advantages and limitations of both production systems across the entire process chain, including feedstock selection, upstream processing, fermentation strategies and downstream recovery, with particular attention to low-cost saline substrates and waste-derived feedstocks and their potential implications for process sustainability and economic feasibility.

## 2. Polyhydroxyalkanoates (PHAs)

Polyhydroxyalkanoates (PHAs) are bio-based and biodegradable linear aliphatic polyesters accumulated intracellularly by microorganisms as carbon and energy reserves. They consist of (R)-hydroxyalkanoic acid monomers and are commonly classified according to monomer chain length as short-chain-length PHAs (scl-PHAs, C3–C5), medium-chain-length PHAs (mcl-PHAs, C6–C14), and long-chain-length PHAs (lcl-PHAs, >C14) [9,10]. Poly(3-hydroxybutyrate) (PHB), the most extensively studied scl-PHA, is highly crystalline and therefore relatively stiff and brittle, whereas mcl-PHAs generally show lower crystallinity and more flexible or elastomeric behaviour [10,11,12]. Copolymerization further modifies these properties; for example, incorporation of 3-hydroxyvalerate into PHB to form poly(3-hydroxybutyrate-co-3-hydroxyvalerate) (PHBV) generally reduces crystallinity and improves flexibility [13,14]. Consequently, PHA properties are determined primarily by monomer composition, side-chain structure, molecular weight, and crystallinity rather than by classification as a PHA alone [2,15,16].

Molecular weight is particularly relevant because it affects melt processability, mechanical strength, and degradation behaviour. PHAs commonly exhibit molecular weights in the range of approximately 2 × 10^5^–3 × 10^6^ Da, although the final value depends on the producing strain, substrate, cultivation conditions, and recovery method [17,18,19]. Salinity can also influence PHA molecular weight, although the response appears to be strain- and process-dependent [20,21]. The underlying mechanisms have not been fully elucidated but may involve salinity-dependent changes in PHA-synthase activity, precursor availability, intracellular polymer turnover, or chain-termination reactions. These findings should not be generalized across PHA-producing microorganisms because molecular weight is also affected by strain, substrate, cultivation phase, and recovery method. In saline systems, salinity may influence polymer characteristics indirectly by altering microbial growth, precursor allocation, monomer composition, and PHA-synthase activity. However, the available evidence does not indicate a uniform relationship between increasing salinity and polymer molecular weight. Residual salts can additionally affect biomass processing, polymer purification, and the reliability of subsequent physicochemical characterization. Studies of saline PHA production should therefore report molecular weight and dispersity, monomer composition, crystallinity, thermal behaviour, and residual salt content together with polymer yield [8,22]. The resulting range of material properties supports applications in packaging, agriculture, and biomedicine, as well as the production of high-value monomeric precursors [15,23,24,25,26,27,28,29,30].

## 3. Microbial PHA Production in Saline Conditions

Polyhydroxyalkanoate (PHA) accumulation is a widespread and conserved trait across Bacteria and Archaea, reported in hundreds of species spanning more than 70 genera. PHAs are stored as hydrophobic cytoplasmic inclusions (carbonosomes) composed of a polyester core surrounded by granule-associated proteins, including phasins, which regulate polymer synthesis, mobilization, and structural stability [31]. Under conditions of carbon excess coupled with limitation of essential nutrients (e.g., nitrogen, phosphorus, or oxygen), microorganisms redirect surplus carbon toward intracellular PHA synthesis [30]. Polyhydroxyalkanoate biosynthesis is tightly integrated with central carbon metabolism, including glycolysis, the tricarboxylic acid (TCA) cycle, β-oxidation, de novo fatty acid synthesis, amino acid catabolism, and autotrophic pathways. Acetyl-CoA serves as the principal metabolic node linking these pathways to PHA formation [32,33]. These metabolic routes are highly conserved among PHA-producing microorganisms, including halophilic and halotolerant bacteria and archaea, which have evolved additional physiological adaptations to cope with osmotic stress while maintaining efficient carbon storage metabolism [6,34,35]. scl-PHAs are synthesized via a conserved three-step enzymatic pathway involving β-ketothiolase (PhaA), acetoacetyl-CoA reductase (PhaB), and PHA synthase (PhaC). Initially, two acetyl-CoA molecules condense to form acetoacetyl-CoA (PhaA), which is subsequently reduced to (R)-3-hydroxybutyryl-CoA (PhaB). This hydroxyacyl-CoA serves as the direct substrate for polymerization by PhaC, yielding intracellular polyester granules. These reactions are typically encoded by the phaCAB operon and are highly conserved across bacterial species [33,36]. In halophilic bacteria, particularly members of the genus *Halomonas*, this pathway is similarly conserved and supports high PHA accumulation under saline and nutrient-limited conditions, demonstrating that osmotic adaptation and carbon storage metabolism are closely interconnected [6,37,38]. Hydroxyacyl-CoA precursors originate from multiple metabolic routes depending on substrate availability. Carbohydrate metabolism via glycolysis predominantly generates acetyl-CoA, favoring the production of scl-PHAs (C3–C5 monomers), as observed in organisms such as *Cupriavidus necator* [36,39]. Under nutrient-limited, carbon-excess conditions, reduced TCA cycle flux leads to acetyl-CoA accumulation and its redirection toward PHA biosynthesis, facilitating carbon storage and relieving CoA-mediated inhibition of β-ketothiolase [33]. Fatty acid β-oxidation constitutes an alternative precursor-supplying pathway, producing diverse intermediates such as enoyl-CoA and (R)-3-hydroxyacyl-CoA. These intermediates enable the synthesis of both scl- and mcl-PHAs, depending on substrate composition [40]. For instance, *Pseudomonas* species grown on fatty acids typically produce mcl-PHAs containing C6-C14 monomers, resulting in polymers with increased flexibility [39]. Similarly, several halotolerant *Halomonas* species efficiently metabolize fatty acids, glycerol, molasses, and other industrial by-products under saline conditions, highlighting their remarkable metabolic versatility and their suitability for the valorization of saline waste streams [6,34,37]. De novo fatty acid synthesis provides a third route, wherein elongation intermediates (hydroxyacyl-ACP/CoA) are redirected toward PHA production. This pathway primarily contributes to mcl-PHA formation, yielding polymers with lower crystallinity and enhanced elastomeric properties compared to scl-PHAs [41,42].

PHA synthases are classified into four major groups according to their subunit composition and substrate specificity. Class I synthases consist of a single PhaC subunit and preferentially polymerize short-chain-length monomers, as exemplified by *Cupriavidus necator*. Class II synthases, commonly found in *Pseudomonas* species, preferentially utilize medium-chain-length monomers. Class III synthases are heterodimeric PhaC/PhaE complexes found in several photosynthetic bacteria and haloarchaea, whereas Class IV synthases are distinct PhaC/PhaR complexes characteristic of Bacillus species. Both Class III and Class IV synthases mainly polymerize short-chain-length monomers [2,24]. Notably, the extremely halophilic archaeon *Haloferax mediterranei* possesses a Class III PHA synthase and is distinguished by its ability to synthesize poly(3-hydroxybutyrate-co-3-hydroxyvalerate) directly from structurally unrelated carbon sources without precursor supplementation, a characteristic that enhances its industrial attractiveness [34,35,43]. Overall, PHA composition is governed by the interplay between metabolic flux distribution and synthase specificity. Carbohydrate-based metabolism typically supports scl-PHA synthesis via acetyl-CoA, whereas fatty acid metabolism and lipid biosynthesis expand precursor diversity, enabling mcl-PHA production. This metabolic flexibility allows microorganisms to tailor polymer structure in response to environmental and nutritional conditions [33,39]. Polyhydroxyalkanoates (PHAs) are synthesized by a broad diversity of Gram-positive and Gram-negative bacteria as well as several archaeal species, highlighting the widespread distribution of this storage metabolism across phylogenetically distinct microorganisms [33]. While conventional PHA producers such as *Cupriavidus necator*, *Pseudomonas putida*, *Bacillus* spp., and *Azotobacter vinelandii* have long served as model production platforms, increasing attention has recently been directed toward microorganisms inhabiting saline and hypersaline environments. The continuous search for novel and more efficient PHA-producing microorganisms has stimulated extensive bioprospecting efforts across diverse natural environments. Besides the cultivation dependent approaches [44], large-scale genomic datasets have considerably expanded our understanding of microbial diversity, enabling the identification of previously unexplored taxa with biotechnological potential. In particular, microorganisms inhabiting extreme environments have attracted growing interest because adaptation to harsh ecological conditions is often associated with unique metabolic traits that can be exploited for industrial PHA production [45]. Among extreme ecosystems, saline and hypersaline environments have emerged as particularly promising reservoirs of novel PHA-producing microorganisms. Comparative analyses of PhaC gene distribution have revealed that bacterial and archaeal PHA synthases are especially abundant in non-marine saline and alkaline habitats [45,46]. Soda lakes represent one of the best-characterized examples of these ecosystems, combining high salinity, alkaline pH, abundant inorganic carbon in the form of carbonates and bicarbonates, and frequently limited nitrogen availability. These environmental conditions favour the selection of metabolically versatile microorganisms capable of efficient carbon storage through PHA accumulation, which could potentially serve as alternatives to established native PHA producers such as *Cupriavidus necator* and recombinant production chassis such as *Escherichia coli*. The ecological success of these microorganisms is closely linked to the multifunctional role of PHAs. Beyond serving as intracellular carbon and energy reserves, PHAs contribute to microbial adaptation by supporting redox homeostasis, protecting against oxidative stress, mitigating damage caused by dehydration and osmotic fluctuations, and improving survival during nutrient limitation [13,47]. Under moderate osmotic stress, growth limitation and altered carbon or redox balance may favour carbon allocation to PHA in some strains. Conversely, at higher salinity, the increased energetic demand for ion homeostasis and compatible-solute synthesis may reduce the carbon and reducing equivalents available for polymer accumulation. Salinity may also influence PHA synthesis through changes in the expression or activity of pathway enzymes, including PhaC, although these responses remain strain- and condition-dependent.

## 4. PHA Production by Halophilic and Halotolerant Microorganisms

### 4.1. Haloarchaea

Haloarchaea represent an important group of saline PHA producers, with *Haloferax mediterranei* being the most extensively investigated example. This organism is particularly attractive because it naturally synthesizes PHBV from structurally unrelated substrates, including molasses, whey, crude glycerol, and volatile fatty acids (VFAs), without requiring extensive precursor supplementation [48]. This metabolic versatility provides an important advantage for the valorization of inexpensive industrial and agro-food residues. Salinity strongly influences both growth and PHA accumulation in haloarchaea. Elevated salt concentrations impose osmotic stress and require cells to maintain intracellular homeostasis through ion regulation and metabolic reorganization. These responses modify cellular energy requirements and carbon-flux distribution and can favour the diversion of assimilated carbon toward PHA biosynthesis, particularly under nutrient-limited conditions [5,30]. In this context, PHA functions not only as a carbon and energy reserve but also as part of a broader physiological response associated with redox balancing, osmotic adaptation, and cellular homeostasis. The relationship between salinity and PHA production is generally non-linear. Increasing salinity may enhance intracellular polymer accumulation while simultaneously reducing biomass formation because of the energetic cost associated with osmoadaptation. In *Hfx. mediterranei*, increasing sodium chloride (NaCl) concentration has been reported to redirect carbon flux from extracellular polysaccharide synthesis toward intracellular PHA storage, resulting in PHA contents exceeding 70% of cell dry weight (CDW) under high-salinity conditions [48]. Thus, an optimal salinity window is required in which osmotic stress is sufficient to stimulate storage metabolism without severely compromising growth. Carbon-source selection is similarly important for PHBV production by *Hfx. mediterranei*. The organism can metabolize whey, crude glycerol, molasses, and mixed VFAs, and significant incorporation of 3-hydroxyvalerate (3HV) can be achieved without extensive supplementation with dedicated precursors [48,49]. VFAs such as acetate, propionate, butyrate, and valerate are particularly attractive because they can serve both as carbon sources and as metabolic precursors influencing copolymer composition. Crude glycerol is another promising feedstock because *Hfx. mediterranei* can tolerate impurities such as residual methanol, salts, and fatty acids, supporting its integration into waste-valorization and biorefinery processes [30,50]. Although *Haloferax mediterranei* remains the most extensively investigated haloarchaeal platform for PHA production, PHA accumulation has also been reported in other members of the Halobacteria, including species of *Haloferax*, *Haloarcula*, *Halogeometricum*, and *Natrinema* isolated PHA-producing haloarchaea belonging to *Natrinema altunense* and *Haloterrigena jeotgali* species evaluating their ability to use inexpensive carbon sources such as sugar wastes [51,52,53].

### 4.2. Halophilic Bacteria

Halophilic bacteria offer several advantages over conventional mesophilic PHA producers from a bioprocess perspective. Their ability to grow under elevated salinity substantially reduces the risk of microbial contamination, enabling cultivation under minimally sterile conditions. Saline cultivation can also permit the direct use of seawater and saline waste streams while facilitating downstream processing through osmotic cell disruption, reducing freshwater consumption and simplifying polymer recovery [48,54].

Among currently available bacterial production platforms, species of the genus *Halomonas* are the most extensively investigated. These microorganisms tolerate high salinity, with some strains exhibiting optimal growth at NaCl concentrations exceeding 100 g NaCl L^−1^. *Halomonas boliviensis* efficiently utilizes carbohydrates and can accumulate more than 80% PHB under nitrogen-limited fed-batch cultivation, whereas *Halomonas bluephagenesis* has emerged as a robust industrial chassis capable of high-cell-density cultivation under non-sterile conditions [37,54,55]. Other species, including *Halomonas elongata*, *Halomonas campisalis*, and *Halomonas hydrothermalis*, can synthesize PHB and PHBV from diverse substrates while maintaining growth across broad salinity ranges. A major advantage of *Halomonas* species is therefore the combination of salt tolerance, metabolic versatility, and relatively flexible nutritional requirements. Beyond *Halomonas*, members of the genus *Vibrio* represent another group of naturally saline-adapted bacteria with potential for PHA production. Early screening of marine isolates demonstrated that several native *Vibrio* strains were capable of intracellular PHB accumulation from diverse carbon sources, with one isolate reaching approximately 41% of cell dry weight when cultivated in medium containing 40 g NaCl L^−1^ [56,57,58,59,60]. More recently, native *Vibrio alginolyticus* strains isolated from salt-field environments were shown to synthesize PHB from a range of sugars and organic acids. In the wild-type (WT) strain *Vibrio alginolyticus*, glycerol was identified as a favorable carbon source in a defined synthetic medium. Optimization of NaCl concentration and cultivation temperature increased the PHB titer from 1.87 to 5.08 g L^−1^. Supplementation with propionate additionally enabled PHBV production [61]. Similarly, the marine halophile *Vibrio proteolyticus* has demonstrated substantial PHB accumulation and the capacity to synthesize PHBV when propionate is supplied as a 3HV precursor [62]. These observations highlight the potential of native *Vibrio* spp. as rapidly growing, substrate-versatile PHA producers adapted to saline cultivation. Nevertheless, their development as industrial production hosts remain less advanced than that of *Halomonas*, and careful strain selection is required because some *Vibrio* species, including *V. alginolyticus*, are recognized as opportunistic pathogens [61].

The capacity to utilize inexpensive and waste-derived substrates further strengthens the potential of halophilic bacteria for PHA production. *Halomonas* strains have been investigated for polymer production from sugarcane molasses and soybean flour, fruit waste, food waste [63], and waste frying oil [21]. Simple carbohydrates such as glucose, fructose, sucrose, and starch hydrolysates nevertheless remain among the most efficient substrates because they can be readily converted into central metabolic intermediates required for PHA biosynthesis. Accordingly, *H. boliviensis* and *H. bluephagenesis* frequently accumulate approximately 70–80% PHB on glucose-rich media, while *Halomonas halophila* has been reported to reach polymer contents approaching 90% of cell dry weight [20,39,64,65]. Low-cost and waste-derived substrates are receiving increasing attention as a means of improving process economics. Molasses, starch-processing residues, lignocellulosic hydrolysates, spent coffee grounds, lipid-rich wastes, waste frying oils, and crude glycerol have all been investigated as potential feedstocks [30]. Several *Halomonas* species can efficiently convert waste oils into PHBV copolymers, while co-feeding sugars with odd-chain organic acids can promote 3HV incorporation and thereby modify polymer flexibility and processability [57,66]. The ability of *V. alginolyticus* to efficiently utilize glycerol further illustrates the potential of saline-adapted bacteria for the valorization of inexpensive carbon sources [67]. Waste-derived substrates nevertheless present additional challenges because their composition may vary considerably among batches and they can contain inhibitory compounds, including furfurals, phenolics, and organic acids [68]. Salinity and carbon-source utilization are closely interconnected. Under osmotic stress, a fraction of assimilated carbon and reducing equivalents must be allocated to compatible-solute synthesis, ion homeostasis, and cellular maintenance rather than biomass or polymer formation. Consequently, substrates that efficiently support energy generation, growth, and osmoadaptation such as glucose, glycerol, and mixed VFAs are particularly attractive for saline fermentation systems [69]. Moderate salinity levels, commonly in the range of approximately 30–100 g NaCl L^−1^ for many moderately halophilic production strains, often provide a favorable compromise between growth and polymer accumulation [39,54]. However, the optimum salinity is strongly strain-dependent and should not be generalized across halophilic taxa.

At excessively high salinity, the energetic burden of osmoadaptation can reduce substrate uptake, enzymatic activity, and biomass formation even when intracellular polymer content remains comparatively high. Maximum volumetric PHA production may therefore occur at intermediate rather than extreme salinity, reflecting a balance between the physiological benefits of saline adaptation and the metabolic costs of maintaining osmotic homeostasis [70]. This relationship is also illustrated by *V. alginolyticus*, for which optimization of NaCl concentration substantially influenced both growth and PHB production [67]. Another halophilic bacterium of interest is *Salinivibrio costicola*, which has been reported to maintain PHB accumulation over a broad salinity range. PHB contents of approximately 30–55% have been observed between 30 and 150 g NaCl L^−1^ [71], further demonstrating the diversity of naturally halophilic PHA-producing bacteria. Collectively, halophilic bacteria display substantial variability in biomass formation, intracellular polymer accumulation, substrate utilization, and productivity. *Halomonas* currently represents the most developed bacterial platform because of its combination of high PHA accumulation, metabolic versatility, and compatibility with not sterile saline fermentation, whereas native *Vibrio* spp. and other marine halophiles broaden the available physiological and metabolic diversity. Their principal advantage over conventional low-salinity producers lies not only in their capacity to tolerate elevated salt concentrations but also in the possibility of integrating non-sterile cultivation, seawater use, saline waste-stream valorization, and simplified downstream processing. However, optimal performance generally depends on maintaining salinity within a strain-specific range that balances osmoadaptation, growth, substrate conversion, and PHA accumulation.

### 4.3. Halotolerant Bacteria

Halotolerant bacteria constitute a complementary group of PHA-producing microorganisms capable of maintaining growth and polymer accumulation over a relatively broad range of salt concentrations without requiring high salinity for optimal growth. This physiological flexibility is particularly advantageous for the valorization of saline or compositionally variable waste streams, where fluctuations in ionic strength may adversely affect conventional production strains. Compared with obligate halophiles, halotolerant bacteria can generally operate under less restrictive saline conditions while retaining the capacity to withstand transient increases in osmotic pressure, thereby providing greater flexibility in process design. Moderate increases in salinity may favour the redirection of carbon toward intracellular PHA storage, whereas excessive salt concentrations progressively increase the energetic requirements associated with ion homeostasis, compatible-solute accumulation, and other osmoadaptive mechanisms. Consequently, the relationship between salinity, biomass formation, and PHA accumulation is often non-linear, and the salinity level that maximizes polymer production does not necessarily coincide with that supporting optimal growth. Several members of the genus *Bacillus* illustrate the potential of halotolerant bacteria for PHA production under saline conditions. A particularly relevant example is *Bacillus cereus* NT-3, a halotolerant strain isolated from high-salinity leachate that maintained substantial PHA production without significant changes at NaCl concentrations up to 50 g L^−1^. The strain was also able to utilize volatile fatty acids derived from the acidogenic fermentation of food waste, highlighting its potential for coupling saline tolerance with the valorization of inexpensive and compositionally complex waste-derived substrates [72,73]. However, the potential scale-up of *B. cereus* would require strain-specific assessment of virulence and toxin production, together with validated containment, biomass-inactivation and effluent-treatment procedures, which have not yet been specifically evaluated in the available PHA-production studies. Similarly, a marine isolate identified as *Bacillus subtilis* SG1 exhibited broad salt tolerance and retained PHA-producing capacity under saline cultivation, yielding a PHA titer of approximately 1.52 g L^−1^ in modified M9 medium containing 50 g NaCl L^−1^ [74,75]. Together, these studies indicate that halotolerant *Bacillus* spp. can retain PHA-producing capacity at salinities that would impose considerable stress on many conventional production hosts, making them attractive candidates for saline effluents and waste streams characterized by fluctuating salt concentrations. A particularly interesting response to salinity has been reported for *Pseudomonas fluorescens* Bar1, a native mcl-PHA-producing strain. Under NaCl-induced osmotic stress, PHA accumulation increased by 5.75-fold at an osmotic potential of −2 bar compared with unstressed conditions and was approximately 1.8-fold higher than under an equivalent osmotic stress generated with PEG-6000 [76]. These results suggest that the stimulation of PHA accumulation was not solely attributable to reduced water availability and that the ionic nature of the stress may also influence intracellular carbon allocation. Moreover, the predominance of mcl-PHA synthesis in *Pseudomonas* broadens the relevance of salinity-induced PHA accumulation beyond the short-chain-length polymers more commonly associated with *Bacillus* spp. Thus, osmotic stress may function not only as an environmental constraint but, within an appropriate range, also as a process variable capable of modulating intracellular carbon allocation and polymer production. The response of these halotolerant microorganisms can be usefully contrasted with that of *Cupriavidus necator*, one of the most established bacterial platforms for industrial PHA production. Although *C. necator* is not generally classified as a halophilic or strongly halotolerant bacterium, moderate NaCl supplementation can influence its PHA-producing metabolism [77,78] evaluated NaCl concentrations ranging from 3.5 to 15 g L^−1^ and demonstrated that controlled salt addition could improve PHA production, whereas increasing salinity beyond the favorable range progressively impaired process performance. The comparison with *Bacillus* spp. and *P. fluorescens* therefore highlights an important distinction between salt-induced stimulation of PHA accumulation and intrinsic halotolerance. In *C. necator*, moderate osmotic stress can be exploited to enhance polymer production, but its useful salinity window remains comparatively restricted; in naturally halotolerant strains, PHA biosynthesis can be maintained over a broader range of osmotic conditions. Overall, halotolerant bacteria broaden the spectrum of native hosts potentially suitable for PHA production from saline and variable-salinity feedstocks. Their principal advantage does not necessarily lie in growth at extreme salt concentrations, but rather in their ability to preserve metabolic activity and polymer synthesis despite fluctuations in osmotic conditions. Within this group, *Bacillus* spp. combine environmental robustness with the capacity to utilize waste-derived carbon sources, whereas *P. fluorescens* provides a particularly interesting example of salt-stimulated mcl-PHA production. Comparison with *C. necator* further emphasizes that the effect of salinity on PHA biosynthesis is highly strain-specific: moderate osmotic stress may enhance carbon storage, whereas beyond a strain-dependent threshold, the increasing energetic costs of osmoadaptation compete with biomass formation and polymer synthesis. Defining this optimal salinity window is therefore an important consideration when selecting and operating microbial hosts for PHA production from saline waste streams.

### 4.4. Engineered Strains for Saline PHA Production

Metabolic and genome engineering have significantly expanded the performance and versatility of saline PHA-production systems. While strain selection, salinity optimization, and substrate choice exploit metabolic properties naturally present in halophilic and halotolerant microorganisms, engineering approaches enable deliberate modification of cellular metabolism. *Halomonas* has become the principal model platform for this strategy within the framework of next-generation industrial biotechnology. Major engineering objectives include redirecting central carbon metabolism toward polymer synthesis, expanding the usable salinity range, improving genetic stability, applying genome-scale metabolic models to strain design, tailoring polymer composition, and reducing downstream recovery costs.

Table 1 summarizes representative PHA-producing haloarchaea, halophilic and halotolerant bacteria, and engineered strains, together with their reported salinity conditions, substrates, fermentation strategies, and production performance.

#### 4.4.1. Modification of the Salinity-Tolerance Window

Engineering osmolyte metabolism has enabled *Halomonas* strains to operate at substantially lower salinity. Deletion of *ectB*, which blocks ectoine synthesis, was identified as a key modification supporting low-salinity growth in *H. bluephagenesis* [83]. Combining this modification with adaptive laboratory evolution, dedicated production modules and process optimization generated an iterative SSM strain series. The fifth-generation strain SSM141 reached 76 g L^−1^ CDW with 86% PHB content and a 39% glucose-to-PHB conversion rate under only 5 g NaCl L^−1^ [84]. The improved low-salinity growth observed after *ectB* disruption may be explained by at least three hypotheses: reduced resource allocation to ectoine biosynthesis and consequent carbon-flux redistribution; altered regulation of osmotic sensing and compatible-solute transport; or compensatory mutations acquired during adaptive laboratory evolution. Because this phenotype resulted from the combined effects of ectB deletion, adaptive laboratory evolution, and process optimization, it cannot be attributed exclusively to the suppression of ectoine synthesis.

#### 4.4.2. Carbon-Flux Redirection and Polymer Tailoring

**Figure 1:**
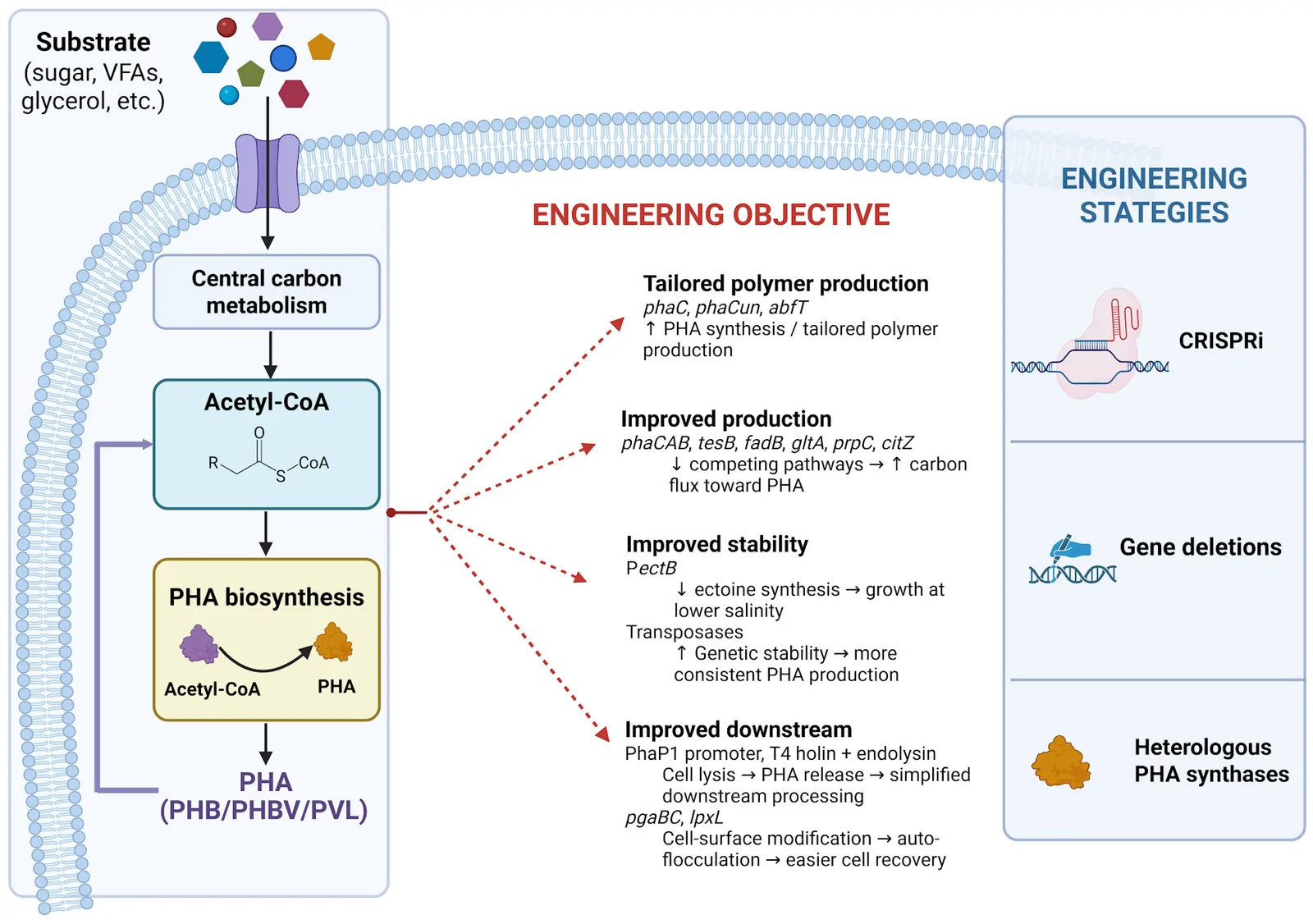
Schematic overview of representative metabolic engineering strategies for enhanced PHA production. The scheme illustrates the connection between central carbon metabolism and PHA biosynthesis, highlighting engineering objectives including tailored polymer production, improved production, enhanced stability, and improved downstream processing. Representative strategies include CRISPR interference (CRISPRi), gene deletions, and the introduction of heterologous PHA synthases. The genes and engineering interventions shown are representative examples and do not constitute a comprehensive representation of the metabolic network. Abbreviations: VFAs, volatile fatty acids; CoA, coenzyme A; PHA, polyhydroxyalkanoates; PHB, poly(3-hydroxybutyrate); PHBV, poly(3-hydroxybutyrate-co-3-hydroxyvalerate); PVL, poly(δ-valerolactone); CRISPRi, CRISPR interference.

Engineered *H. bluephagenesis* strains, including ZS19, ZS49, and TDH4A1B5P, have achieved intracellular PHA contents exceeding 75% CDW and biomass concentrations approaching or exceeding 100 g L^−1^ [80,81,85,86]. The development of self-regulated PHA synthase expression systems and targeted pathway engineering has further enabled biomass concentrations above 120 g L^−1^ and intracellular PHA contents exceeding 80% CDW. These performances approach those of conventional industrial microorganisms while retaining the contamination resistance and reduced sterilization requirements of saline cultivation. A major engineering strategy involves suppressing pathways that compete with PHA synthesis and redirecting metabolic flux toward desired polymers (Figure 1). In *H. bluephagenesis*, co-expression of the phaCun gene, encoding the broad-substrate-specificity PHA synthase PhaC**_BP-M-CPF4_**, and the CoA-transferase gene abfT enabled the biosynthesis of poly(δ-valerolactone) (PVL). Deletion of the endogenous *phaCAB* operon, together with removal of *tesB* and the β-oxidation gene *fadB*, suppressed competing 3-hydroxybutyrate formation to below 2 mol%, resulting in near-homopolymeric poly(δ-valerolactone) production [87]. CRISPR interference (CRISPRi) provides an alternative means of redirecting carbon flux without permanently deleting potentially important genes. In *Halomonas* sp. TD01, repression of the citrate synthase gene *gltA* increased PHB content by approximately 8%, whereas repression of *prpC* increased the 3HV fraction of PHBV to approximately 13% [88]. Although *Halomonas* remains the dominant engineering chassis, comparable strategies have been applied to other saline producers, particularly *Hfx. mediterranei* and *Vibrio* species. In *Hfx. mediterranei*, engineering has focused on eliminating competing carbon sinks and redirecting metabolic flux toward PHBV. Deletion of an EPS-biosynthesis gene cluster eliminated EPS production, resulting in lower broth viscosity, improved dissolved-oxygen availability and reduced foaming, while increasing PHBV production by approximately 20% without impairing growth [89]. Simultaneous CRISPRi repression of the citrate synthase genes *citZ* and *gltA* increased PHBV titer by 76.4%, from 1.78 to 3.14 g L^−1^, while chromosomal integration of the guide-RNA cassette increased productivity by 165% [90]. In addition to redirecting carbon towards PHBV, suppression of EPS formation may decrease mixing and aeration constraints in viscous high-salinity media. Engineering of *Vibrio* species is less mature but has generated promising results. In *V. natriegens*, base editing was used to identify native PHB-pathway genes, which were subsequently overexpressed to improve PHB production from non-sterile crude glycerol [91]. The engineered strain exhibited an approximately 166-fold increase in PHB content relative to the wild type, reaching about 35% CDW. In *V. alginolyticus* LHF01, co-expression of the PHB-biosynthesis operon with an amylase enabled direct starch conversion. The engineered strain more than doubled the PHB titer and consumed 20 g L^−1^ of insoluble corn starch within 6–7 h, producing 4.32 g L^−1^ PHB [61]. Engineering substrate-utilization pathways may therefore broaden the range of feedstocks available for saline fermentation and reduce the need for preliminary substrate hydrolysis. However, these advantages require further validation using real waste-derived feedstocks and larger-scale cultivation. Genome-scale metabolic models have also been employed to identify targets associated with improved PHA synthesis in saline chassis. Models developed for *H. elongata* and *H. campaniensis* identified PHA-synthase expression, glycolysis, fatty-acid metabolism and nitrogen-source utilization as potential determinants of polymer production [92,93].

#### 4.4.3. Genetic Stability During Prolonged Cultivation

Genetic stability represents another important requirement for industrial scale-up [84]. The enzyme-constrained genome-scale metabolic model Halo-ecGEM was used to investigate the relationship between endogenous transposases and batch-to-batch variability in *Halomonas bluephagenesis* [84]. Deletion of eight transposases produced more stable growth and more consistent PHB, PHBV and poly(3-hydroxybutyrate-co-4-hydroxyvalerate) (P34HB) production across repeated fermentations, the practical benefit of this modification is improved batch reproducibility and a lower probability of productivity loss during repeated or prolonged cultivation thereby supporting the development of more robust microbial platforms for industrial PHA production.

#### 4.4.4. Morphology- and Lysis-Assisted PHA Recovery

Cell morphology and lysis have also become engineering targets because downstream recovery can account for approximately 30–60% of PHA production costs. Engineering interventions in *H. bluephagenesis* enlarged cells and intracellular PHA granules and modified the cell surface through interventions including deletion of *pgaBC* and *lpxL* [83]. These modifications promoted auto-flocculation, allowing cells to settle and potentially reducing dependence on centrifugation. A complementary self-lysis strategy was developed by placing T4-phage holin and endolysin under the control of the PHA-responsive PhaP1 promoter [94]. This system triggered cell rupture after PHA-granule accumulation. The engineered process imposed a PHB-yield cost below 9.4%, was demonstrated at scales ranging from 7 to 5000 L, eliminated lysozyme use, reduced processing time and water consumption by 26% and 30.7%, respectively, and increased extracted PHB purity from 96.1% to 99.2%. These modifications provide direct downstream benefits by facilitating biomass separation and reducing enzymatic treatment, processing time and water consumption. Nevertheless, tightly regulated expression is essential to prevent premature lysis, loss of viable biomass or release of cellular material during the production phase.

## 5. Mixed Microbial Cultures (MMCs)

Mixed microbial cultures (MMCs) have emerged as a promising alternative to pure cultures for polyhydroxyalkanoate (PHA) production, particularly when low-cost, heterogeneous waste streams are used as feedstocks [95]. Their metabolic diversity enables the conversion of complex substrates and adaptation to fluctuations in salinity, pH, nutrient availability, and feedstock composition. Moreover, MMC processes generally require less stringent sterility and strain-maintenance procedures than pure-culture systems, potentially reducing operating costs. However, their dynamically changing community composition can compromise process reproducibility and requires careful control of the ecological and operational selection pressures. A major advantage of MMC-based processes is their capacity to valorize municipal, agro-industrial, and food-processing residues, including saline wastewaters and seafood-processing effluents. Complex organic feedstocks are commonly subjected to acidogenic fermentation to produce VFAs, which are preferred precursors for PHA biosynthesis. Early studies demonstrated PHA production from fermented olive mill wastewater, cheese whey, and sugarcane molasses [96,97,98], whereas more recent research has extended this approach to integrated biorefineries based on dairy by-products, food-processing residues, and other agro-industrial side streams [99,100]. Similar strategies have been applied to saline agro-food residues and seafood-processing effluents, coupling wastewater treatment with polymer production [99,101]. Under optimized conditions with VFA-rich substrates, enriched MMCs commonly accumulate PHA at 50–65% of CDW [95,102,103]. Salinity is a major ecological filter in MMC systems. During enrichment under saline conditions, halophilic and halotolerant PHA-storing microorganisms progressively become dominant, whereas non-adapted activated-sludge communities can be strongly inhibited. For example, a marine-sludge consortium enriched at approximately 30 g NaCl L^−1^ became dominated by members of the *Rhodobacteraceae* family and accumulated up to 84.1 wt% PHA; valerate supplementation produced copolymers containing up to 63% 3HV. Conversely, food-waste-derived MMCs showed improved PHA accumulation at 2.5 g L^−1^ NaCl, but substrate uptake and storage declined at 5–15 g L^−1^ NaCl. These findings indicate that gradual acclimation, rather than abrupt salt exposure, is essential for establishing stable and productive halotolerant communities [99,104,105]. MMC-based PHA production generally comprises three stages: acidogenic fermentation of the feedstock, enrichment of PHA-storing microorganisms, and polymer accumulation [5]. Acidogenic fermentation, frequently performed in continuous stirred-tank reactors (CSTRs), converts carbohydrates, proteins, and other organic compounds into a more homogeneous VFA-rich stream. The resulting VFA profile influences polymer composition: even-carbon VFAs primarily favour poly(3-hydroxybutyrate) formation, whereas odd-carbon VFAs promote 3HV incorporation and the synthesis of poly(3-hydroxybutyrate-co-3-hydroxyvalerate) [5,106,107]. Enrichment is most commonly achieved in sequencing batch reactors (SBRs) through feast–famine selection, also termed aerobic dynamic feeding. Alternating periods of carbon excess and depletion favour microorganisms that rapidly take up VFAs and store them intracellularly as PHA, progressively outcompeting non-storing populations [102,103]. At salinities close to that of seawater (approximately 30 g NaCl L^−1^), this regime selects osmotolerant marine taxa and generally reduces community diversity. Members of the *Rhodobacteraceae* family have accounted for more than 90% of some enriched communities and achieved PHA contents exceeding 80% of CDW [99]. Other taxa identified in saline PHA-producing consortia include *Pseudorhodobacter* spp. and *Neptunomonas phycophila*, which possess traits associated with PHA biosynthesis and osmotic-stress adaptation [108,109]. Despite the selective pressure imposed by salinity, the long-term stability of saline MMCs may be affected by phage–host interactions, as phages can promote the turnover of dominant populations in PHA-producing communities, and evidence from hypersaline brine microbiomes indicates that viral predation contributes to microbial community structure and dynamics even under extreme salinity conditions [110,111]. Community assembly and polymer productivity are jointly governed by salinity, organic loading rate (OLR), solids retention time (SRT), nutrient supply, and feeding regime. Changes in these parameters can shift the dominant taxa, substrate preferences, storage capacity, and monomer composition. In particular, valerate-rich feeds and elevated OLRs have been associated with increased 3HV incorporation, demonstrating the close relationship between operating conditions, microbial ecology, and polymer properties [99,108,109,112]. Reactor configuration is therefore critical to process stability. Continuous systems may provide high volumetric productivity and facilitate integration into existing infrastructure, but they are vulnerable to biomass washout when the growth rate of PHA-storing microorganisms is lower than the dilution rate. This risk increases with variable waste streams containing residual non-fermented substrates that support competing, non-storing populations. SBR and fed-batch configurations are consequently preferred because they maintain feast–famine selection and permit temporal control of growth and storage phases [5,16,106,107]. Biomass-retention or immobilization strategies can further decouple SRT from hydraulic retention time (HRT), increase cell density, and prevent the loss of slowly adapting halophilic populations. Together with feed control based on pH or dissolved oxygen, these strategies can improve the robustness and scalability of saline MMC-based PHA production [5,99,109].

### Saline Wastes for PHA Production from MMCs

The economic feasibility of MMC-based PHA production largely depends on the availability of low-cost feedstocks. In saline systems, particular attention has been devoted to organic waste streams generated by seafood-processing, fish-canning, dairy, and agro-food industries, which are often characterized by high salt concentrations and substantial organic loads. Following acidogenic fermentation, these substrates can be converted into volatile fatty acid (VFA)-rich streams suitable for the enrichment of halophilic and halotolerant PHA-producing communities [99]. Among the most extensively investigated substrates are seafood-processing effluents, including cooked mussel wastewater, fish-canning brines, and other saline fishery by-products. These streams are particularly attractive because they combine high organic content with salinity levels that naturally favor the selection of halotolerant microbial communities. A representative example is cooked mussel processing wastewater, a saline effluent containing proteins, carbohydrates, and fermentation intermediates. In one study, anaerobic fermentation was coupled with a modified sequencing batch reactor (SBR) operation incorporating a settling phase after the feast period, thereby promoting the removal of non-PHA-accumulating microorganisms and reducing interference from residual organic matter. This strategy increased PHA accumulation from approximately 40 to 60 wt% of CDW and raised hydroxyvalerate content from 17% to 30%, demonstrating the synergistic effect of pretreatment and process optimization [113]. A more comprehensive example involved a three-stage process comprising acidogenic fermentation (CSTR), aerobic enrichment (SBR), and accumulation (FBR) applied to pre-acidified cooked mussel wastewater containing approximately 17–18 g NaCl L^−1^. Protein-to-VFA conversion was significantly enhanced through NaHCO_3_ supplementation and pH control, increasing conversion efficiency from 10.3% to 69.2%. Subsequent modification of the enrichment strategy improved biomass retention and promoted selection of PHA-storing populations. As a result, intracellular PHA accumulation increased from 8.8% to 41.5% of CDW, highlighting the importance of integrated pretreatment and operational optimization for saline protein-rich wastewaters [101]. Beyond seafood-processing residues, other saline agro-food wastes have also shown considerable potential for PHA production. Recently, pickle brine waste (PBW), containing approximately 15 g L^−1^ NaCl, was successfully converted into PHAs by halotolerant MMCs, achieving polymer accumulations of up to 74 wt%. Microbial analyses revealed the enrichment of halotolerant genera including *Neomegalonema*, *Paracoccus*, and *Rhodobacter*, demonstrating the suitability of saline vegetable-processing residues as alternative feedstocks for PHA production [114]. Additional saline agro-food residues, including cheese whey, fish-canning brines, tuna-processing effluents, and brine sludges, have also been investigated following acidogenic fermentation. Fermented cheese whey supported PHA accumulations of approximately 56% of CDW under near-seawater salinity conditions, whereas fish- and tuna-processing brines rich in acetate required careful control of ammonium concentrations to avoid excessive biomass growth at the expense of polymer storage. Pretreatment approaches such as pH adjustment, settling, and enzymatic hydrolysis therefore play a crucial role in improving substrate suitability and enhancing PHA storage efficiency in saline MMC systems [115]. When saline wastes contain substantial amounts of carbohydrates, proteins, or lipids, pretreatment is generally required to increase VFA availability and improve process performance. Carbohydrate-rich residues typically undergo hydrolysis or fermentation, while protein-rich wastes require proteolytic degradation to facilitate subsequent acidogenesis. Under saline conditions, pretreatment should also minimize inhibitory compounds that may negatively affect microbial activity and polymer accumulation. For example, the combination of mild alkaline pretreatment and saline adaptation enabled halophilic MMCs to efficiently utilize corn stover hydrolysate and achieve approximately 40% PHA accumulation [5,22]. Beyond agro-food residues, fatty wastes and crude glycerol have emerged as promising substrates because their metabolism generates high levels of acetyl-CoA, a key precursor for PHA biosynthesis. Halotolerant MMCs are particularly advantageous in these systems due to their ability to tolerate oily and impure waste streams that may inhibit conventional microorganisms. For example, an MMC enriched at 5 g NaCl L^−1^ accumulated nearly 70% PHA from emulsified waste frying oil supplemented with propionate, producing a copolymer containing 40–50% 3HV. Likewise, crude glycerol from biodiesel production has been successfully utilized by marine sludge MMCs under 10–30 g NaCl L^−1^ conditions, yielding approximately 40–50% PHB accumulation despite the presence of impurities [22,50,116]. Saline industrial wastewaters, seawater, and brines can therefore serve not only as process media but also as valuable carbon sources following acidogenic conversion into VFAs. Acidified fish and seafood-processing wastes have supported PHA accumulations of approximately 40–60% with substantial 3HV incorporation, while techno-economic assessments suggest that coupling saline wastewater treatment with PHA production may significantly reduce production costs. These findings highlight the potential of saline MMC processes as integrated waste valorization strategies within circular bioeconomy frameworks [22,101,115].

## 6. Downstream Processing of PHA Produced Under Saline Conditions

Downstream processing remains a major technical and economic constraint in PHA production because the intracellular polymer must be separated from non-PHA cellular material (NPCM) without compromising purity, molecular weight, or thermal properties. Depending on the microorganism, biomass composition, and recovery method, downstream operations may account up to 50% of total PHA production costs [117]. Conventional approaches include mechanical disruption, digestion with sodium hypochlorite, and extraction with chlorinated solvents. Although these methods can provide high recovery and purity, their requirements for energy, hazardous chemicals, and solvent recovery reduce the environmental benefits of PHA production and may cause oxidative or hydrolytic degradation of the polymer [118,119]. Saline production systems require additional consideration of residual salts, brine management, and corrosion-resistant equipment, but they can also enable recovery strategies based on the osmotic sensitivity of salt-adapted cells. However, the amount and quality of available evidence differ considerably between pure cultures and MMCs. Hypotonic lysis and integrated salt recovery have been experimentally investigated for selected halophilic pure cultures, whereas recovery from saline MMC biomass remains poorly documented.

### 6.1. Experimentally Validated Recovery from Halophilic Pure Cultures

Hypotonic lysis is the most distinctive downstream option for strongly halophilic microorganisms. Haloarchaea maintain a high intracellular ionic concentration and possess cell envelopes that become unstable when cells are transferred abruptly from concentrated brine to low-ionic-strength water. The resulting osmotic shock releases intracellular PHA granules and reduces the need for intensive mechanical disruption or concentrated chemical digestion [120]. Nevertheless, lysis efficiency depends on the microorganism, cultivation salinity, biomass pretreatment, and composition of the cell envelope; consequently, washing, surfactant treatment, or solvent polishing is commonly required to remove proteins, membrane lipids, and other residual NPCM. *Haloferax mediterranei* is the best documented example [120]. Early studies demonstrated the recovery of PHBV from this haloarchaeon through osmotic disruption followed by additional purification [49,120]. Osmotic lysis was subsequently applied to *H. mediterranei* cultivated on rice-based ethanol stillage, followed by SDS and organic-solvent purification. The same study recovered and reused 96% of the salts from the spent cultivation medium, showing that salt management can potentially be integrated with polymer recovery rather than treated solely as an effluent-disposal problem. Nevertheless, the energy, water and material requirements of the complete salt-recovery loop were not comprehensively quantified.

A recent TEA of PHBV production by *Haloferax mediterranei* compared solvent extraction with solvent-free osmotic-shock recovery. The estimated minimum selling prices were approximately USD 4.20 kg^−1^ for solvent extraction and USD 3.50 kg^−1^ for osmotic-shock recovery, indicating an economic benefit from simplified downstream processing. Nevertheless, even the osmotic-shock configuration remained approximately 35% above the study’s target price of USD 2.60 kg^−1^. Feedstock price, fermentation yield and PHA-recovery efficiency were identified as major economic determinants. Salt recycling substantially decreased salt demand and saline purge generation, although its effect on the final selling price was comparatively modest. The analysis also emphasized that saline-wastewater disposal costs are location-dependent and that assumptions concerning wash-water use, salt recycling and large-scale solid–liquid separation require experimental validation [121].

A more recent process was developed by [122] for the halophilic bacterium *Halomonas halophila*. Hypotonic exposure was combined with dilute SDS at elevated temperature to disrupt the cells and solubilize hydrophobic contaminants. Under optimized conditions, the recovered PHA exceeded 99 wt% purity and the recovery yield approached quantitative values. The authors also precipitated SDS with KCl and proposed its regeneration, thereby addressing surfactant removal from the aqueous effluent. This study provides experimental evidence that osmotic disruption can also be applied to a bacterial halophile, although heat and detergent were required. Its applicability to other halophilic bacteria cannot yet be generalized because cell-envelope composition and osmotic sensitivity are strain-dependent. After cell disruption, residual NPCM may be removed by mild detergent washing or solvent extraction. Dimethyl carbonate (DMC), ethyl acetate, and other non-halogenated solvents have been investigated as alternatives to chloroform because they combine PHA solubilization with lower toxicity and potential solvent recycling [123]. However, solvent performance is influenced by PHA composition, crystallinity, biomass moisture, and residual salt content. Entrained salts and water may impair solvent–biomass contact, modify phase separation and decrease polymer purity. Nevertheless, the effects of salt concentration and ionic composition on solvent extraction have not been systematically evaluated.

The effects of each treatment on molecular weight should also be verified rather than inferred from recovery and purity alone. In addition, residual salt and solvent concentrations should be measured in the final polymer, particularly when material properties or high-purity applications are considered.

### 6.2. Recovery from Saline Mixed Microbial Cultures: Current Evidence and Transferability

PHA-rich MMC biomass contains cells with different envelope structures together with extracellular polymeric substances, mineral solids, and feedstock-derived residues. Accordingly, downstream schemes generally combine biomass conditioning, NPCM digestion, polymer separation, and washing. These biomass characteristics may affect disruption efficiency, chemical consumption, phase separation and final polymer purity. Moreover, differences in osmotic sensitivity among community members may prevent uniform cell disruption by hypotonic treatment. Although MMC enrichment and accumulation under saline conditions are increasingly reported, recovery studies performed specifically on saline MMC biomass remain limited. Current process design therefore draws mainly on methods validated with non-saline MMCs, whose transferability to salt-rich biomass still requires direct assessment. In non-saline MMC biomass non-halogenated solvent extraction from PHA-rich MMC biomass and identified DMC as a promising alternative to chloroform [123]. Direct DMC extraction preserved polymer molecular weight more effectively than oxidative pretreatment, whereas sodium hypochlorite increased recovery but substantially reduced molecular weight. These results underline the need to select pretreatment intensity according to the required polymer quality. DMC and ethyl acetate may represent candidate solvents for saline MMC biomass. However, this application remains prospective because the effects of residual salts, mineral impurities and extracellular polymeric substances on extraction efficiency, solvent recovery and product purity have not been directly evaluated. In a study conducted with non-saline MMC biomass, laboratory- and pilot-scale MMC biomass with 0.05–1.0 M NaOH, alone or with 0.1% SDS [124]. Mild alkaline conditions provided high recovery and purity, whereas increasing NaOH concentration or extending the treatment promoted polymer loss or molecular-weight reduction. For pilot-scale biomass produced from fermented organic waste, treatment with 0.2 M NaOH and 0.1% SDS for 3 h yielded approximately 80% recovery and 79% purity. The lower performance relative to cleaner laboratory biomass was attributed to the greater content of residual solids. This result highlights the importance of feedstock-derived impurities when translating extraction methods to real waste-based biomass. A further study with non-saline pilot-scale MMC biomass developed a chlorine-free recovery process using biomass produced from fermented sewage sludge [125]. Sequential NaOH and H_2_O_2_ treatment achieved 93 wt% purity and 88 wt% recovery when applied to approximately 200 g of biomass, demonstrating the scalability of aqueous NPCM digestion beyond analytical-scale tests. Ethyl acetate extraction was also investigated as a non-toxic solvent-based route. More recent comparisons of recovery methods applied to MMC biomass derived from waste-activated sludge further demonstrated that treatment conditions must be optimized jointly for recovery, purity and molecular-weight retention [126].

Enzymatic digestion and physical disruption offer additional routes for reducing the use of hazardous solvents and oxidants. Proteases, lysozyme, nucleases, and lipases can selectively digest NPCM, often in combination with low surfactant concentrations, while bead milling and high-pressure homogenization can improve cell disruption [119]. EDTA-assisted microwave treatment has achieved 93.8% recovery and 97.2% purity from recombinant *Escherichia coli*, while preserving a substantially higher PHB molecular weight than sodium-hypochlorite treatment [127]. However, this method has not yet been validated for halophilic pure cultures or saline MMCs and should therefore be considered a prospective option rather than an established saline downstream process.

### 6.3. Salt Management, Reporting Requirements and Research Gaps

Residual salt is a central issue throughout saline downstream processing. Biomass harvesting and washing generate concentrated brines, whereas inadequate desalting may contaminate the polymer and interfere with detergent or solvent recovery. Water consumption must also be considered because repeated freshwater washing can offset the benefits of hypo-osmotic lysis. Integrated salt recovery and reuse, as demonstrated for *H. mediterranei* [128], has the potential to reduce both freshwater demand and saline effluent generation. Equipment exposed to alternating high-salinity and freshwater streams must additionally resist corrosion and salt deposition.

## 7. Comparison Between Pure Cultures and MMCs and Future Perspective

Both pure cultures and MMCs have been successfully employed for PHA production under saline conditions, although they differ in process control, substrate requirements, productivity, polymer consistency, and downstream recovery. The available studies differ substantially in salinity and ionic composition, substrate characteristics, cultivation strategy, reactor configuration, experimental scale, and calculation of performance indicators, limiting direct quantitative comparison between these platforms. Accordingly, the following comparison is qualitative and process-oriented. It aims to identify recurrent characteristics and potential application contexts rather than establish the superiority of either platform. Table 2 summarizes these qualitative tendencies and should not be interpreted as a quantitative benchmark. Halophilic pure cultures generally provide reproducible performance, high intracellular PHA accumulation, and greater control over monomer composition and molecular-weight distribution. Well-characterized strains can also be selected or engineered for the targeted production of PHB, PHBV, and mcl-PHAs. Although conventional pure-culture processes frequently depend on defined media and relatively refined substrates, strongly halophilic production strains may require less stringent sterile conditions because elevated salinity can limit the growth of some contaminant. Nevertheless, maintaining a single dominant population generally requires tighter control of cultivation conditions and feedstock composition.

Saline MMCs offers greater ecological flexibility and can utilize heterogeneous substrates, including seafood-processing effluents, saline agro-food residues, industrial brines, and fermented organic wastes. This versatility may facilitate the use of low-cost feedstocks while integrating PHA production with wastewater treatment and waste valorization. Salinity acts as an additional selective pressure, favoring halophilic and halotolerant PHA-storing populations and suppressing many non-adapted contaminants. However, MMC performance is intrinsically linked to community dynamics. Variations in substrate composition, salinity, nutrient availability, organic loading rate, and feeding regime can modify the abundance and activity of PHA-storing populations, affecting polymer accumulation, monomer composition, and batch-to-batch consistency. Stable operation therefore requires carefully controlled selection pressures and, frequently, prolonged community acclimation. Despite these advantages, direct comparisons between saline pure-culture and MMC-based processes remain difficult because studies frequently differ in salinity, substrate composition, cultivation mode, nutrient limitation, and performance metrics. Consequently, differences in reported PHA content or productivity cannot always be attributed solely to the microbial platform. Establishing standardized experimental and reporting criteria would therefore be important to enable a more robust assessment of the relative performance of pure cultures and MMCs. The two platforms also present different downstream-processing requirements. In halophilic pure cultures, high and relatively uniform intracellular PHA contents reduce the proportion of non-PHA cellular material requiring removal. The two platforms also present different downstream-processing requirements. Pure cultures generally produce more homogeneous biomass, facilitating the optimization of cell disruption and purification. By contrast, MMC biomass may contain microorganisms with different cell-envelope properties, together with extracellular polymeric substances, mineral particles and feedstock-derived residues, which can complicate polymer recovery. However, direct experimental evidence concerning recovery from saline MMC biomass remains limited. In both platforms, biomass desalting, water consumption, brine management, and preservation of polymer molecular weight remain important considerations. From an industrial perspective, these downstream constraints should be considered together with upstream process requirements. Although saline cultivation can reduce contamination risks and enable the use of less-sterile conditions, the accumulation of salts in the biomass and process streams may increase water demand for washing and generate concentrated brines requiring appropriate management. Thus, the potential reduction in sterilization requirements should be evaluated against the additional costs associated with salt removal and wastewater management.

The selection of the production platform should therefore reflect the intended application and process context. Halophilic pure cultures may be favoured when high productivity, reproducible polymer properties, targeted monomer composition, and standardized recovery are required. Saline MMCs are attractive for waste- and wastewater-based processes in which substrate flexibility, resource recovery, and reduced feedstock costs are primary objectives. However, these potential advantages require validation under comparable operating conditions and at relevant experimental scales.

Life-cycle assessments specifically addressing PHA production under saline conditions remain limited. Koller et al., 2013 evaluated PHA production by *Haloferax mediterranei* using whey-derived substrates [129], while Roibás-Rozas et al., 2020 assessed the environmental performance of PHA production from saline industrial wastewaters using mixed microbial cultures [130]. More recently, Wang et al. 2026 examined non-sterile PHB production by *Halomonas* sp. WZQ-1 from biowastes [131]. These studies suggest that the environmental performance of saline PHA processes depends strongly on feedstock characteristics, PHA yield, energy and chemical requirements for substrate pretreatment, and downstream recovery. Therefore, potential benefits associated with reduced sterilization requirements and saline-waste valorization should be evaluated together with freshwater consumption, salt management, brine disposal and polymer-recovery requirements. Additional LCAs based on harmonized functional units, system boundaries and experimentally validated scale-up data are needed before general conclusions regarding environmental sustainability can be drawn.

For pure cultures, future research should evaluate production stability during prolonged or repeated cultivation and determine whether reduced-sterility operation can be maintained at larger scales without loss of productivity or strain stability. Engineered saline chassis should be assessed not only according to PHA accumulation but also in terms of salt requirements, genetic stability, contamination susceptibility, and downstream benefits. For saline MMCs, particular attention should be directed towards long-term community stability under variations in salinity, ionic composition, and waste-feedstock characteristics. The effects of bacteriophages, microbial predators, and seasonal feedstock variation also require investigation. Finally, techno-economic assessment and life-cycle assessment should be conducted using clearly defined and comparable system boundaries. These analyses should include sterilization, salt supply, equipment corrosion, freshwater use, desalting, brine treatment, solvent recovery, and waste management. Geographical availability, seasonal variability, and competing uses of saline resources and waste-derived feedstocks should also be considered. The two platforms provide complementary development routes for saline PHA production rather than representing competing alternatives. Their future implementation will depend on the integration of microbial physiology, process control, standardized performance assessment, and resource-efficient downstream processing.

## Figures and Tables

**Table 1:** Representative PHA-producing microorganisms under different salinity conditions, grouped by microbial category.

Organism	PHA (% CDW)	PHA Titer (g L^−1^)	Productivity (g L^−1^ h^−1^)	Substrate(s)	NaCl (g L^−1^)	Molecular Weight	PHA Composition	Fermentation Strategy/Scale	References
**A. Haloarchaea**									
*Haloferax mediterranei*	70%	19.7	0.2	Distillery Vinasse	200	n.r.	PHBV	Non-sterile shake-flask culture (250 mL)	[30,34,49,50]
	50%	n.r.	0.3	Native Cornstarch	230.4	n.r.	PHBV	pH-stat fed-batch culture in 6 L bioreactor	
	73%	12.2	0.1	Hydrolyzed Whey Permeate	200	n.r.	PHBV	Fed-batch culture in 42 L bioreactor	
	43–57%	0.3–0.7	0.002–0.005	Food Waste-Derived Carboxylates (VFAs)	150.6	n.r.	PHBV	Aerated batch culture (laboratory scale)	
	53.5%	1.57	0.008–0.011	Pure VFA Mixture	150.6	n.r.	PHBV	Aerated batch culture (laboratory scale)	
*Natrinema altunense*	61%	14.8	0.2	Glucose	200	n.r.	PHBV	Batch culture in shake flask (250 mL)	[53]
*Haloterrigena jeotgali*	3.6–25%	0.1	0.001	Glucose	250	n.r.	PHV	Batch culture in shake flask (250 mL)	[52]
	2.9–3.1%	0.004	0.0004	Industrial Sugar Waste	250	n.r.	PHV	Batch culture in shake flask (250 mL)	
*Haloarcula* sp.	34.6%	0.08	0.001	Glucose	290.2	n.r.	PHB	Batch culture in shake flask (250 mL)	[51]
	22.4%	0.06	0.0008	Starch	290.2	n.r.	PHB	Batch culture in shake flask (250 mL)	
*Halogeometricum borinquense*	74%	4.2	0.2	Glucose	200	n.r.	PHBV	Batch culture in shake flask (250 mL)	[30,79]
	50.4%	1.6	0.01	Sugar cane bagasse hydrolysate	200	n.r.	PHBV	Batch culture in shake flask (250 mL)	
**B. Halophilic bacteria**									
*Halomonas boliviensis*	81%	35.4	1.1	Glucose	40.5	1.100 kDa	PHB	Fed-batch culture (laboratory scale)	[54,56,65]
	50–56%	1.7–7.8	0.4	Agricultural Residues	40.5	n.r.	PHB	Batch culture in shake flasks or bioreactors (laboratory scale)	
*Halomonas bluephagenesis*	60.4–82.3%	103.9–60	1.67–1.12	Glucose	10–60	n.r.	PHB	Open/non-sterile fed-batch culture (pilot scale)	[39,54,55,80]
	60–70%	13–24	0.26–0.57	Glucose	60	n.r.	PHB	Two-stage connected open continuous process (laboratory scale)	
	75%	24	0.55	Glucose + Urea	60	n.r.	PHB	Open/non-sterile culture (laboratory scale)	
*Halomonas halophila*	81.5%	4.58	0.06	Glucose	60	793.91 kDa	PHB	Batch culture in shake flask (250 mL)	[20]
	61.95%	2.17	0.03	Spent Coffee Grounds Hydrolysate	60	n.r.	PHB	Batch culture in shake flask (250 mL)	
*Halomonas elongata*	22.81–32%	2.2–2.4	0.02–0.05	Glucose	10–50	n.r.	PHB	Batch culture in shake flask (500 mL)	[58,60]
	55%	2.4	0.02	Glycerol	100	n.r.	PHB	Fed-batch culture in 2 L bioreactor	
*Halomonas campisalis*	45–81%	0.6–1.1	0.02–0.05	Maltose	5–50	1301.4 kDa	PHBV	Single-stage batch culture in shake flasks 250 mL and Scale-up batch fermentation in a 120 L	[57]
*Vibrio alginolyticus*	60.9%	5.0	0.2	Glycerol	50	201 kDa	PHB	Batch culture in shake flask (250 mL)	[67]
*Vibrio proteolyticus*	28.5–54%	0.3–2.7	0.007–0.05	Fructose	50	n.r.	PHB	Batch culture in shake flask (250 mL)	[62]
*Salinivibrio* sp. *TGB10*	81.8%	27.4	0.2	Glucose + Propionate	20.7	295 kDa	PHBV	Fed-batch cultivation in a 5 L bioreactor	[66]
	87.6%	26.3	0.3	Glucose	20.7	344 kDa	PHB	Fed-batch cultivation in a 5 L bioreactor	
**C. Halotolerant bacteria**									
*Bacillus cereus*	67.9%	5.1	0.1	Fructose	5	n.r.	PHB	Batch culture in shake flask (100 mL)	[72,73]
	48.4%	3.1	0.004	Food Waste Acidogenic VFAs Effluent	5–50	n.r.	PHBV	Batch culture in shake flask (250 mL)	
*Bacillus subtilis*	n.r.	1.5	0.02	Glucose	50	n.r.	PHB	Batch culture in shake flask	[74]
*Cupriavidus necator*	70–80%	121–232	0.8–3.1	Glucose/VFA Ethanol	0–5	n.r.	PHB	Fed-batch culture in a 5 L bioreactor	[47,77]
	78%	5.3	0.2	Acetic Acid	9	n.r.	PHB	Fed-batch culture in a 5 L bioreactor	
**D. Recombinant (engineered) bacteria**									
Engineered *H. bluephagenesis* (ZS19)	76–80.7%	19.5–84.6	0.3–2.4	Glucose	30–50	n.r.	PHB	Open non-sterile fed-batch culture in a 7 L bioreactor and Shake-flask batch culture (500 mL)	[81]
Engineered *H. bluephagenesis* (TDH4A1B5P)	68.1–82.3%	43.8–108	0.9–2.8	Glucose	10.5	n.r.	PHB	Open non-sterile fed-batch culture in a 5 L bioreactor and Shake-flask batch culture (500 mL)	[80]
*Halomonas* sp. TD01	80%	64	1.3	Glucose	60	600 kDa (Fed-batch) 340 kDa (Continus)	PHB	Fed-batch and Continus culture in a 6 L bioreactor and Shake-flask batch culture (500 mL)	[37]
*H. campaniensis* LS21	83%	7.8	0.2	Glucose	20.7	n.r.	PHB	Open non-sterile fed-batch culture in a 6 L bioreactor and Shake-flask batch culture (500 mL)	[82]
*Halomonas elongata* A1	90.7%	6.1	0.1	Glucose	50	n.r.	PHB	Culture in a 6 L bioreactor and Shake-flask batch culture (500 mL)	[60]
*Vibrio alginolyticus* LHF01 (*phaBAPC*)	42.1%	4.5	0.25	Soluble Starch	50	n.r.	PHB	Batch culture in shake flask	[61]

CDW = cell dry weight; VFAs = volatile fatty acids; n.r. = not reported; PHBV = poly(3-hydroxybutyrate-co-3-hydroxyvalerate); PHB = poly(3-hydroxybutyrate); PHV = polyhydroxyvalerate.

**Table 2:** Qualitative process-oriented comparison of pure halophilic cultures and saline mixed microbial cultures for PHA production.

Aspect	Pure Cultures (Halophilic)	Mixed Microbial Cultures (MMC)
Microbial control	Very high (defined strain)	Low (variable community)
Reproducibility	High	Medium-low
Polymer composition	Controllable (PHB, PHBV, mcl-PHA)	Variable, less predictable
Substrate flexibility	Medium (requires adaptation)	Very high (complex waste streams)
Substrate cost	Medium	Low
Sterility requirement	No (under saline conditions)	No
Contamination resistance	High (salt-selective environment)	Very high (ecosystem robustness)
Operational robustness	Medium	High
Sensitivity to feed variation	High	Low
Process strategy	Controlled fed-batch	Feast/famine (sequencing batch)
Process development time	Long (strain optimization needed)	Shorter
Industrial scalability	High (already demonstrated)	Still developing
Downstream processing	Easier (homogeneous biomass)	More complex (heterogeneous biomass)
PHA purity	High	Medium
Genetic engineering potential	Yes	No

PHB = poly(3-hydroxybutyrate); PHBV = poly(3-hydroxybutyrate-co-3-hydroxyvalerate); mcl-PHA = medium-chain-length PHA.

## Data Availability

No new data were created or analyzed in this study. Data sharing is not applicable to this article.
